# Blocking the formation of radiation–induced breast cancer stem cells

**DOI:** 10.18632/oncotarget.1992

**Published:** 2014-05-20

**Authors:** Yangyang Wang, Wende Li, Shalin S. Patel, Juan Cong, Nan Zhang, Francesco Sabbatino, Xiaoyan Liu, Yuan Qi, Peigen Huang, Hang Lee, Alphonse Taghian, Jian-Jian Li, Albert B. DeLeo, Soldano Ferrone, Michael W. Epperly, Cristina R. Ferrone, Amy Ly, Elena F. Brachtel, Xinhui Wang

**Affiliations:** ^1^ Division of Surgical Oncology, Department of Surgery, Massachusetts General Hospital, Boston, MA; ^2^ Department of Radiation Oncology, Massachusetts General Hospital, Boston, MA; ^3^ Department of Orthopaedics, Massachusetts General Hospital, Boston, MA; ^4^ Department of Biostatistics Center, Massachusetts General Hospital, Boston, MA; ^5^ Department of Radiation Oncology, University of California, Davis, Sacramento, CA; ^6^ Department of Pathology, University of Pittsburgh, Pittsburgh, PA; ^7^ Department of Radiation Oncology, University of Pittsburgh, Pittsburgh, PA; ^8^ Division of General and Gastrointestinal Surgery, Department of Surgery, Massachusetts General Hospital, Boston, MA; ^9^ Department of Pathology, Massachusetts General Hospital, Boston, MA; ^10^ Harvard Medical School, Boston, MA

**Keywords:** Breast Cancer, Radiation, Cancer Stem Cells, NF-kappaB, Stemness genes

## Abstract

The goal of adjuvant (post-surgery) radiation therapy (RT) for breast cancer (BC) is to eliminate residual cancer cells, leading to better local tumor control and thus improving patient survival. However, radioresistance increases the risk of tumor recurrence and negatively affects survival. Recent evidence shows that breast cancer stem cells (BCSCs) are radiation-resistant and that relatively differentiated BC cells can be reprogrammed into induced BCSCs (iBCSCs) via radiation-induced re-expression of the stemness genes. Here we show that in irradiation (IR)-treated mice bearing syngeneic mammary tumors, IR-induced stemness correlated with increased spontaneous lung metastasis (51.7%). However, IR-induced stemness was blocked by targeting the NF-κB- stemness gene pathway with disulfiram (DSF)and Copper (Cu^2+^). DSF is an inhibitor of aldehyde dehydrogenase (ALDH) and an FDA-approved drug for treating alcoholism. DSF binds to Cu^2+^ to form DSF-Cu complexes (DSF/Cu), which act as a potent apoptosis inducer and an effective proteasome inhibitor, which, in turn, inhibits NF-κB activation. Treatment of mice with RT and DSF significantly inhibited mammary primary tumor growth (79.4%) and spontaneous lung metastasis (89.6%) compared to vehicle treated mice. This anti-tumor efficacy was associated with decreased stem cell properties (or stemness) in tumors. We expect that these results will spark clinical investigation of RT and DSF as a novel combinatorial treatment for breast cancer.

## INTRODUCTION

Adjuvant RT is given to the breast cancer patients after conservation surgery and may be given to the chest wall after mastectomy to achieve better local tumor control thus improving survival of patients [1-3]. However, radioresistance impedes the anti-tumor effects of RT and could be attributed to BCSCs, since it has recently been shown that BCSCs are radioresistant[4, 5]; but radiation also can induce BCSCs, i.e., iBCSCs from nonstem breast cancer cells [6]. In order to significantly improve the efficacy and curability of RT for breast cancer, therefore, novel therapeutic approaches are urgently needed to not only eliminate radioresisitant pre-existing BCSCs, but also block the formation of radiation induced new BCSCs from nonstem BC cells. This study is a pre-clinical effort to develop such a new treatment, for rapid translation into clinical practice, that is scientific sound, simple, safe and economic.

Following the first discovery that mature adult cells can be reprogrammed to become pluripotent stem cells (iPSCs) by introduction of a set of four stemness factors (Oct4, SOX2, KLF4 and MYC) through retroviral transduction [7], it has been shown that cancer stem cells (CSCs) and relatively differentiated cancer cells are subject to bidirectional conversion [8]. Stemness gene-encoded transcription factors (TFs) play a central role in the determination of stem cell states [7], with specific transcriptional networks having essential roles in sustaining the growth and self-renewal of embryonic and neoplastic stem-like cells. The TFs Slug and SOX9 cooperatively determine mammary stem cell states in both normal and malignant cells [9]. Moreover, the overexpression in human cancers of reprogramming TFs correlates with tumor progression and poor prognosis [10]. IR has been shown to reprogram differentiated cancer cells into iBCSCs or liver CSCs through the re-expression or upexpression of the stemness genes Oct4/SOX2/Nanog/KLF4 and SOX2/OCT3/Oct4, respectively [6, 11].

There is compelling evidence showing that the cells expressing elevated levels of intracellular aldehyde dehydrogenase 1 (ALDH1) in human and mouse breast cancer are BCSCs [12, 13]. The ability of enhanced ALDH1 activity in these cells to eliminate genotoxic aldehydes contributes to their radio- and chemo-resistance. Research on the CSC area has been facilitated by the ability to routinely detect and isolate CSCs by flow cytometry analysis using the ALDEFLUOR reagent to measure their ALDH enzymatic activity [6, 14]. Consequently, ALDH activity is widely used as a CSC marker for many malignancies including leukemia, lung, liver, bone, colon, pancreatic, prostate, head and neck, bladder, thyroid, brain, and cervical cancer and melanoma [15]. Moreover, we demonstrated that ALDH1A1 is not only a marker but also a therapeutic target for CSCs [14, 16, 17].

DSF (Brand name: Antabuse) is an irreversible pan-ALDH inhibitor; it inhibits all the currently identified cytosolic and mitochondrial ALDH isoforms [18, 19], resulting in accumulation of, in particular, acetaldehyde, which causes unpleasant effects when alcohol is consumed. As a result, DSF has been an FDA approved drug for treatment of alcoholism since 1951 [20]. Studies have shown that in cells DSF converts to diethyldithiocarbamate (deDTC) and that** two molecules of deDTC bind to one molecule of copper (Cu^2+^) to form the Cu[deDTC]_2_ complex (DSF/Cu) [21-23]. Cu^2+^ is an essential trace element for life [24] as it plays a crucial role in redox reactions and generation of reactive oxygen species (ROS) in human cells [25, 26]. It is known that DSF/Cu is an effective proteasome inhibitor resulting in inhibition of NF-κB [21, 27]. NF-κB is a key TF governing the activation of many genes involved in stress responses (e.g. IR), cell survival, apoptosis, inflammation, and radioresistance [28]. These NF-κB regulated stemness genes include ERBB2 [4], SOX9 [29], MYC [30] and WNT [31]. We have, therefore, investigated using human and mouse BC cell lines and a clinically relevant mouse model whether DSF/Cu can block *in vitro* and *in vivo* the IR-induced conversion of nonstem BC cells into iBCSCs via downregulation of the NF-κB-stemness gene pathway and enhance the efficacy of RT.

## RESULTS

### DSF/Cu effectively depleted pre-existing BCSCs and radiation-induced BCSCs

**Figure 1:**
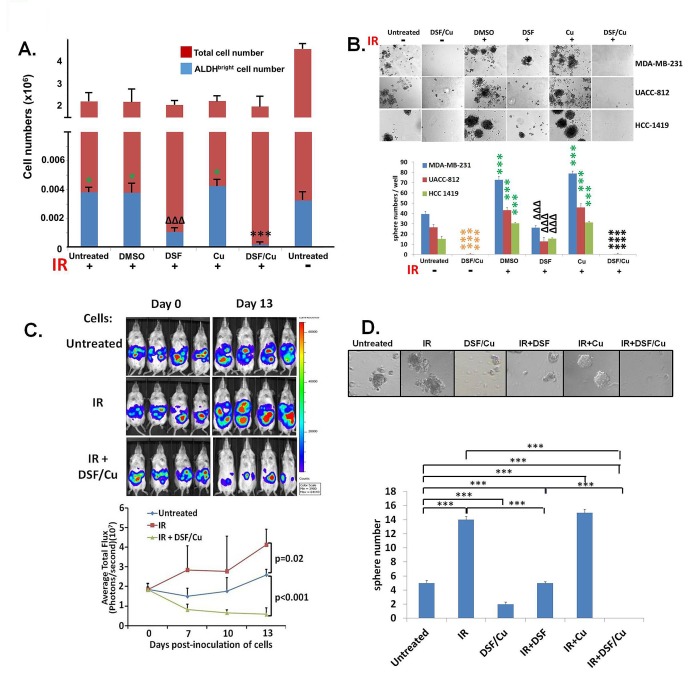
Depletion of BCSCs/iBCSCs by DSF/Cu as measured by decreased ALDH^bright^ cells, mammosphere formation *in vitro* and tumorigenicity *in vivo* Fraction-irradiated (3.75 Gy/day × 5 days) BC cells were cultured for 24 hours, followed by treatment with different agents for an additional 24 hours, as indicated. The total numbers of cell were counted using the Trypan blue exclusion method. The numbers of ALDH^bright^ cells were calculated based on the total cell numbers and % of ALDH^bright^ cells. * indicates P<0.05: IR or IR+DMSO or IR+Cu vs. untreated; *** indicates P<0.001: IR+DSF/Cu vs. any other treated or untreated; ΔΔΔ indicates P<0.001: IR+DSF vs. any other treated or untreated (A). Mammosphere formation assays were setup by seeding cells from fraction-irradiated human BC cell lines and culturing them for 14 days. Pictures were taken and mammospheres were quantified by counting mammosphere numbers per well on day 14. *** indicates *P*<0.001: IR+DMSO or IR+Cu vs. untreated; *** indicates *P*<0.001: IR+ DSF/Cu vs. any other treated or untreated except DSF/Cu; ΔΔΔ indicates P<0.001: IR+DSF vs. any other treated or untreated; *** indicates P<0.001: DSF/Cu vs. any other treated or untreated except IR+DSF/Cu (B). Three groups of 6 week old female NOD/SCID mice (n=4) were injected intraperitoneally (i.p.) with either untreated MDA-MB-231-luc cells (Untreated) or fraction-irradiated MDA-MB-231-luc cells and then cultured for 24 hours followed by an additional 24 hours of culture with (IR+DSF/Cu) or without (IR) treatment with DSF/Cu. Each group was administered 2x10^6^ cells in 25μL serum- and drug-free RPMI 1640 + 25μL Matrigel/mouse. Tumor growth was monitored in each mouse every 3-4 days by whole animal bioluminescent imaging (BLI) and tumor burden, quantitated as photons/second. Increased tumorigenicity in IR treated and lack of tumorigenicity in IR+DSF/Cu treated, compared to untreated cells, were observed. P values for comparison between groups are shown (C). Using diethylaminobenzaldehyde (DEAB), an inhibitor of ALDH1/3 isoforms, to establish the baseline fluorescence as the gating reference standard of ALDH^neg^ population, FACS sorted MDA-MB-231 ALDH^neg^ cells were fraction-irradiated and then cultured for 24 hours, followed by treatment with different agents for an additional 24 hours, as indicated for mammosphere formation assays. *** indicates P<0.001(D). All experiments (presented in A, B, D) were performed in duplicate and repeated twice, and the means ± SD are shown.

Based on compelling evidence showing that elevated ALDH activity in human and mouse BC cells is a marker for BCSCs and iBCSCs [6, 12-14], in this study we have identified these cells by flow cytometry analysis of BC cells as ALDH^bright^ cells, namely those ALDH^+^ cells with twice the mean fluorescence intensity (MFI) of the bulk ALDH^+^ cell population. We detected an increased percentage of BCSCs *in vitro* following fractionated irradiation (3.75 Gray (Gy)/day × 5 days) of BC cell lines MDA-MB-231 (2.4 fold), SUM149 (1.4 fold) and UACC-812 (4.6 fold) (Supplementary Fig. S1A). Within a range of doses of fractionated irradiation (1-5 Gy/day × 5 days), increased ALDH^bright^ cells were detected in BC cell lines (Supplementary Fig. S1B). The increased percentage of BCSCs was caused by an increase in the absolute number of BCSCs accompanied by a 50.5% decrease in total cell number in irradiated cells vs. untreated cells, which indicates that IR induced the formation of new BCSCs or iBCSCs (Fig. 1A). The stem cell functional properties of these BCSCs and iBCSCs were further supported by *in vitro* formation of mammospheres (Fig. 1B) and increased tumorigenicity of the *in vitro* irradiated BC cells compared to untreated BC cells in mice (Fig. 1C). Treatment of cells with DSF/Cu effectively depleted pre-existing (before IR) BCSCs and iBCSCs (together referred to as BCSCs/iBCSCs) (Fig. 1A, B, C), including those induced by IR from nonstem ALDH^neg^ cells, as evidenced using this cells isolated by fluorescence-activated cell sorting *in vitro* (Fig. 1D). In contrast, DSF/Cu or IR and DSF/Cu did not exhibit toxicity on normal human mammary epithelial cells as measured by cell growth and apoptosis assays (Supplementary Fig. S1C).

### The combination of *in vitro* treatment with IR and DSF/Cu induced more potent apoptosis of BC cells than either single treatment alone

We reasoned that the depletion of BCSCs/iBCSCs by DSF/Cu could be due to a combination of mechanisms: 1) induction of apoptosis and/or 2) obstruction of conversion of nonstem BC cells into iBCSCs. It is known that DSF/Cu is a potent inducer of apoptosis of BC cells through, at least partially, upregulation of the pro-apoptotic ROSmitogen-activated protein kinases (MAPK) pathway [27]. We found evidence consistent with this, as p38 MAPK was upregulated and additionally, we found activation of the pro-survival AKT was inhibited in human BC UACC-812 cells treated with a combination of IR and DSF/Cu. These data strongly suggest increased apoptosis in BC cells exposed to this combinatorial treatment vs. DSF/Cu alone (Supplementary Fig. S2).

### DSF/Cu blocked the IR-induced stemness via downregulation of the NF-κB-stemness gene pathway *in vitro*

**Figure 2:**
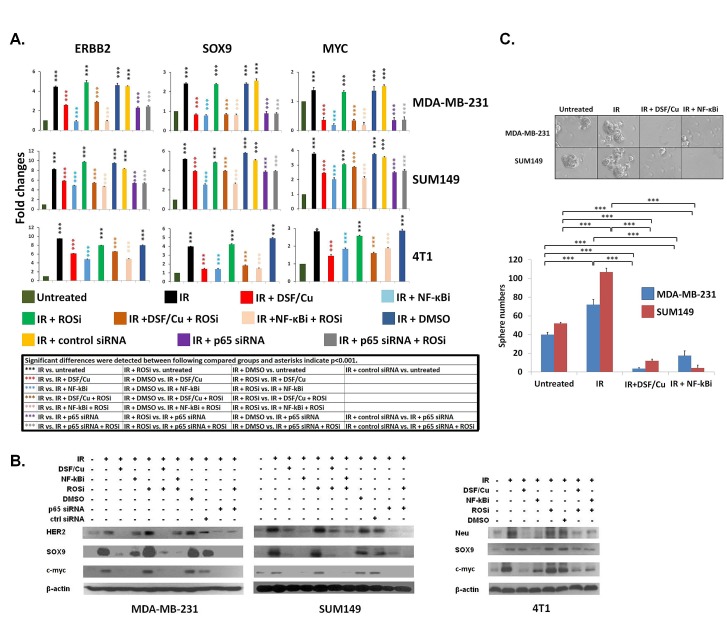
Inhibition by DSF/Cu of IR-induced stemness gene upregulation at the mRNA and protein levels of BC cells *in vitro* Non-transfected BC, BC cells transfected with NF-κB p65 siRNA or control siRNA were fraction-irradiated (3.75 Gy/day × 5 days). Non-transfected, fraction-irradiated BC cells were cultured for 24 hours, followed by treatment with different agents as indicated for an additional 24 hours. siRNA transfected fraction-irradiated BC cells were cultured for 24 hours, followed by treatment with NAC for an additional 24 hours. The expression of ERBB2, SOX9 and MYC (genes named by HUGO gene database) was analyzed by real-time quantitative (q) RT-PCR. Gene expression values obtained from treated cells were compared with those obtained from untreated cells, which were standardized to a value of 1. The results of the comparison were expressed in terms of fold change ± SD and *P* values are shown (A). Treated cells were lysed and analyzed by Western blotting for expression of HER2/Neu, SOX9 and c-myc (proteins named by NCBI protein database) (B). Mammosphere formation assays were performed with cells treated as indicated. Pictures were taken on day 10 (SUM149) and on day 14 (MDA-MB-231). *** indicates P<0.001(C). The concentrations of NF-κBi and ROSi used were1 μM and 10 mM, respectively. All of the experiments were performed in duplicate and repeated twice, and the means ± SD are shown.

As DSF/Cu has been shown to be an effective proteasome inhibitor, that can result in inhibition of NF-κB activity [21, 27]. We determined that DSF/Cu was able to markedly inhibit IR-induced upregulation of NF-κB (Supplementary Fig. S3). Studies have also shown that NF-κB plays a major role in regulating the expression of the stemness genes ERBB2 [4], SOX9 [29], MYC [30], and WNT [31]. In this regard, we found potential NF-κB binding sites in the promoter regions of WNT3 for human and mouse (Supplementary Table S1). However, the role of NF-κB in determining stem cell fates of irradiated BC cells has never been investigated. Using two human and one mouse BC cell lines, we detected significant increases *in vitro* of stemness gene expression of ERBB2, SOX9, and MYC at the mRNA and protein levels in irradiated cells (Fig. 2A, B and Supplementary Table S2). *In vitro* treatment of irradiated cells with DSF/Cu reduced the expression of these stemness genes at the mRNA as well as protein levels (Fig. 2A, B and Supplementary Table S2). Additionally, we showed that the inhibitory effect of DSF/Cu on stemness gene expression was NF-κB dependent, as treatment with either the NF-κB inhibitor(NF-κBi) IMD-0354 or siRNA knockdown of NF-κB (p65) expression displayed similar stemness inhibition as measured by gene expression and mammosphere formation (Fig. 2A-C). In contrast, treatment with the ROS inhibitor (ROSi) N-Acetyl-L-cysteine (NAC) did not. ROS did not seem to play a role in modulating the NF-κB- stemness gene pathway, since the ROS inhibitor did not impact the ability of either DSF/Cu, IMD-0354 or the NF-κB p65 siRNA to inhibit the expression of stemness genes (Fig. 2A, B).

### DSF blocked IR-induced stemness *in vivo* resulting in inhibition of both primary tumor growth and spontaneous lung metastasis

**Figure 3:**
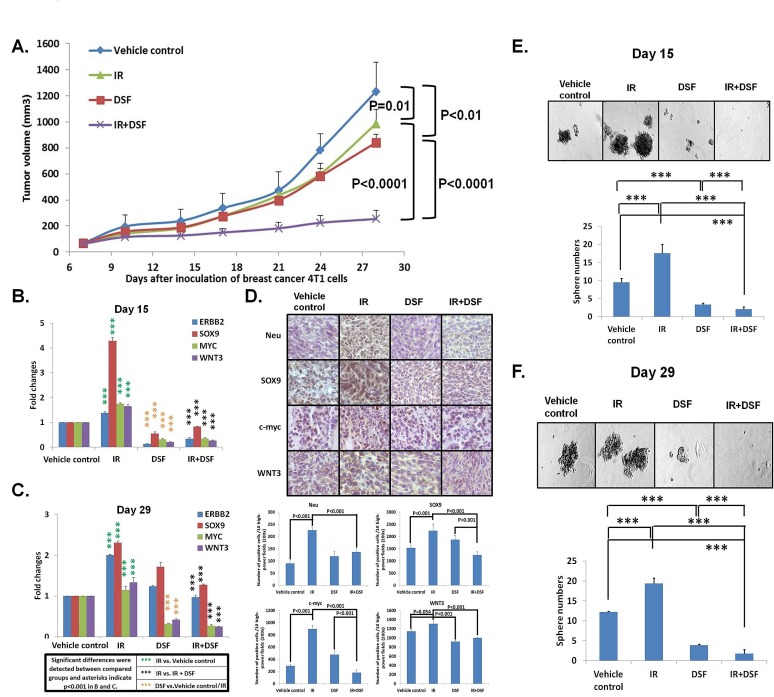
IR + DSF is more potent than either DSF or IR alone in targeting 4T1 tumors in mice Mouse 4T1 cells were injected subcutaneously (s.c.) into the right hind leg of 28 mice. On day 7, when the tumors reached 5 mm in diameter, mice were assigned in a stratified randomized manner to 4 groups (n=7 per group) and the treatments were initiated. Group 1 (Vehicle control) was treated orally with the vehicle control olive oil. Group 2 (IR) was administered a single dose of IR (20Gy) on day 10. Group 3 (DSF) was treated orally with DSF once daily for 8 days. Group 4 (IR+DSF) was treated orally with DSF, once daily for 8 days and mice were administered a single dose of IR on day 10. Tumor sizes were monitored twice per week. Mean tumor volumes of each group ± SD and *P* values for comparison between groups are shown (A). On day 15, two mice from each group were sacrificed and their tumors (Day 15 tumors) were collected for analysis of intratumoral levels of stemness gene expression. Total RNA was extracted from fresh 4T1 tumors and analyzed for mRNA of stemness genes by quantitative real-time qRT-PCR. Gene expression values obtained from treated tumors were compared with those obtained from untreated tumors, which were standardized to a value of 1. The results of the comparison were expressed in terms of fold change. The experiments were performed in duplicate and the means of fold change ± SD and P values are shown (B). The remaining 5 mice from each group were sacrificed on day 29 and tumors (Day 29 tumors) were collected for analysis of stemness genes using the same method as described above. The experiments were performed in duplicate and the means of fold change ± SD and *P* values are shown (C). Tumors from all mice collected on both days 15 and 29 were immunohistochemically (IHC) stained for protein products of stemness genes. Staining results of Neu, SOX9, c-myc and WNT3 were evaluated and expressed as positive cells in 10 high-power (200x) fields.P values for comparison between groups are shown (D). Day 15 tumors (n=2 per group) were digested to test intratumoral BCSCs/iBCSCs using mammosphere formation assays. The experiments were performed in duplicate and the means ± SD are shown. *** indicates P<0.001 (E). Day 29 tumors (n=5 per group) were digested for analysis of intratumoral BCSCs/iBCSCs using the same method as described above. The experiments were performed in duplicate and the means ± SD are shown. *** indicates P<0.001 (F).

**Figure 4:**
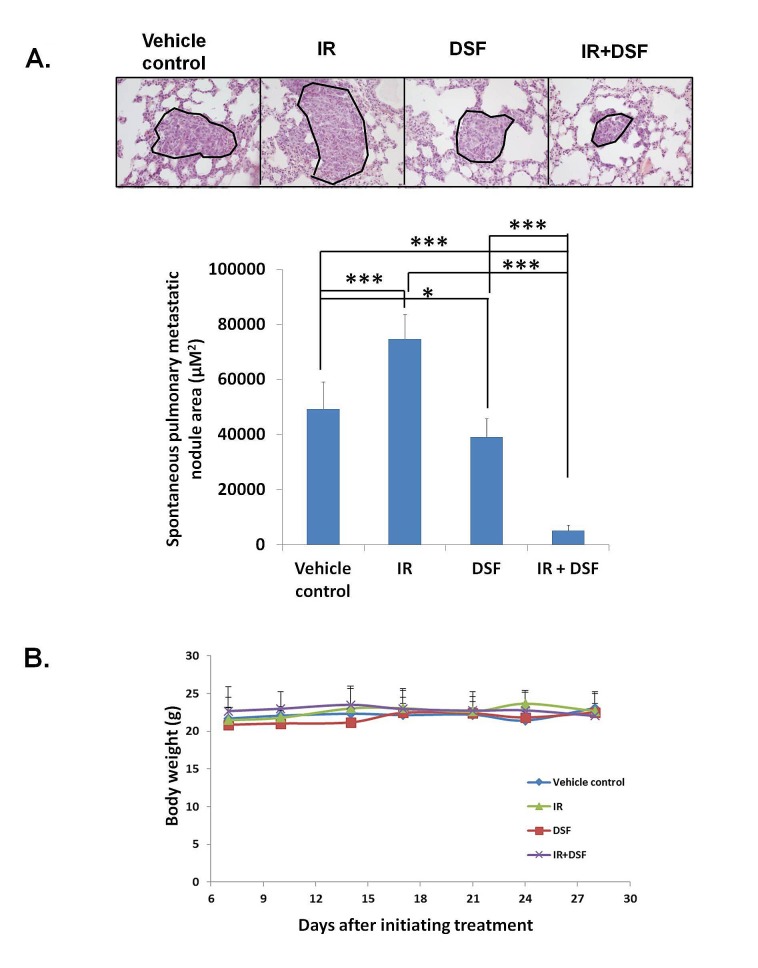
IR+DSF shows potent inhibition of spontaneous lung metastasis counteracting the incremental effect of IR As described in Fig. 3, at the time of sacrifice (on day 29), lungs were harvested from the mice (n=5 per group) and formalin-fixed, paraffin-embedded lung tissues were H&E stained. Representative images are shown at a 200x magnification. The areas of spontaneous pulmonary metastatic nodules in each section were quantified from 10 randomly selected high-power 200x fields. Means ± SD are shown. *** indicates P<0.001 and *indicates P<0.05(A). Whole body weight of each mouse, a marker for toxicity, was measured twice per week. Means ± SD are shown (B).

To determine the impact of IR on stemness in primary breast tumors, and whether DSF/Cu can counteract IR-induced stemness *in vivo*, we used the 4T1 cell line-derived syngeneic BALB/c mouse tumor model, which closely resembles human breast cancer and forms spontaneous lung metastases [32]. Consistent with our *in vitro* findings (Fig. 2A, B), IR increased stemness genes ERBB2, SOX9, and MYC and WNT3 mRNA (1.21-4.52 fold) and protein expression (Fig. 3B-D, Supplementary Table S3). Additionally, the number of mammospheres formed from tumors obtained from IR-treated mice was higher than the number obtained from tumors of mice treated with vehicle, as measured using tumors obtained from these groups of mice one day after the treatment was completed (day 15) and at the end of the experiment (day 29). The increase at day 15 was 61.0%, and at day 29, it was 84.2% (Fig. 3E, F). Although IR yielded a modest reduction in primary tumor volume compared to the vehicle control (20.1%) (Fig. 3A), strikingly, the high level of IR-induced stemness correlated with a 51.7% greater nodular area of spontaneous lung metastases compared to the vehicle control (Fig. 4A). However, we found that the combination of IR and DSF was significantly more effective at suppressing primary tumor growth compared to IR alone (74.2%), DSF alone (71.1%) or vehicle (79.4%) treated mice on day 29 (Fig. 3A). It should be noted that in contrast to *in vitro* experiments, that because a high level of endogenous Cu^2+^ exists in tumor environments, exogenous Cu^2+^ was not needed in these *in vivo*-based experiments for DSF efficacy [21, 33]. The higher potency anti-tumor effect of the combinatorial therapy of the mice was associated with a marked reduction of stemness gene expression and mammosphere formation in primary tumors compared to that of mice treated with either IR or DSF alone or vehicle (Fig. 3B-F). On day 15, the combination of IR and DSF already resulted in a reduction of stemness mRNA expression (4.13-6.25 fold) and mammospheres (87.2%) compared to tumors treated with IR alone (Fig. 3B, D, E, Supplementary Table S3). This effect was sustained until day 29, when the tumor diameter of control mice reached the upper allowable limit of 1.5 cm (Fig. 3C, D, F, Supplementary Table S3). The decreased expression of stemness genes (1.92-5.25 fold) was directly related to the functional properties of BCSCs, as noted by the markedly reduced ability of the treated tumor cells to form mammospheres (87.2%) (Fig. 3C, D, F, Supplementary Table S3). Relative to mice treated with vehicle, DSF-treated non-irradiated mice also exhibited a reduction of stemness gene expression (1.88-6.24 fold on day 15; 0-2.73 fold on day 29) and decreased mammosphere formation (60.2% on day 15; 64.8% on day 29) (Fig. 3B-F and Supplementary Table S3). Clearly, the combination of IR and DSF eliminated the majority of BCSCs/iBCSCs *in vivo*. Consequently, the size of spontaneous lung metastatic nodules was reduced 89.6% in DSF-treated irradiated mice and 20.4% in DSF treated non-irradiated mice, compared to mice treated with vehicle (Fig. 4A). In sharp contrast, as previously noted, there was a 51.7% increase in the size of spontaneous lung metastatic nodules in irradiated mice compared to mice treated with vehicle (Fig. 4A). Toxicity was not observed in the mice enrolled in this study using body weight as an indicator of drug-induced toxicity (Fig. 4B) [34].

## DISCUSSION

RT uses high-energy radiation to kill cancer cells and shrink tumors and is one of the major treatments for breast cancer and other types of cancer with curative or palliative intent [35]. However, radioresistant pre-existing BCSCs, and as our paper describes, the presence of radiation-induced BCSCs are challenging for a beneficial outcome of RT as BCSCs are the root of cancer initiation, progression, and metastasis [36, 37]. In an effort to significantly improve the efficacy of RT for breast cancer, we investigated and identified a way to target radioresistant pre-existing BCSCs and block the formation of radiation induced BCSCs with DSF/Cu. Our study shown here not only confirmed independently the finding of radiation induces BCSCs, first reported by F Pajonk's group [6], but also identified the role of the NF-κB-mediated stemness gene pathway in the generation of IR-induced stem cells from nonstem BC cells. In addition, we found that IR had only a modest effect on controlling primary tumor growth at the same time promoting spontaneous lung metastasis in a syngeneic mouse model system. This surprising and provocative finding was confirmed repeatedly in *in vivo* experiments using the same mouse model (Fig. 4A, B and data not shown). The significant increase in lung metastasis in IR treated mice compared to vehicle treated mice is likely attributed to IR-induced stemness (Fig. 3B-F). Nevertheless, it is noteworthy that the inhibition of primary tumor growth may also be a contributing factor to significant decrease in lung metastasis in IR and DSF treated mice compared to those treated with either IR or DSF alone (Fig. 3A, Fig. 4A). When comparing IR and DSF with DSF alone, it was shown that the products of the four stemness genes at the mRNA level in day 15 tumors were comparable in both groups but markedly reduced compared to those detected in tumors of mice treated with IR. However, in day 29 tumors, mRNA expression of c-myc and WNT3 were reduced in both groups of mice relative to the IR group, but ERBB2 and SOX9 mRNA levels were significantly reduced only in the tumors of mice treated with IR and DSF (Fig.3B, C). Of the four stemness gene products analyzed in day 15 and 29 tumors at the protein level, the expression of SOX-9 and c-myc, but not ERBB2 and WNT3, was significantly lower in the mice treated with the IR and DSF than DSF alone. Furthermore, the number of mammospheres of tumors treated with IR and DSF, however, was significantly lower than that of tumors treated with DSF alone (Fig. 3E, F). Therefore, it seems that SOX9 and c-myc might play more dominant roles than ERBB2 and WNT3 in determining tumor stemness in this tumor model.

Through investigation of the mechanism underling the inhibition of stemness gene expression, we found that DSF/Cu and NF-κB inhibitors, which both result in a blockade of NF-κB signaling, prevented the formation of iBCSCs. The downregulation of NF-κB signaling by DSF/Cu or the NF-κB inhibitor or NF-κB p65 siRNA knockdown significantly blocked IR-induced expression of stemness genes ERBB2, SOX9, MYC and WNT3 (Fig. 2A, B). As a result of these events, markedly decreased or depleted BCSCs/iBCSCs were detected using *in vitro* and *in vivo*-based assays, namely ALDH activity as a marker (Fig. 1A), mammosphere formation (Fig. 1B, D, Fig. 2C), tumorigenicity *in vivo* (Fig. 1C), mammosphere formation of digested primary tumor cells (Fig. 3E, F) and spontaneous lung metastasis (Fig. 4A). The information from our study contributes to our understanding of the bidirectional conversion between iBCSCs and nonstem BC cells and introduces the novel concept of blocking the formation of iBCSCs as a result of IR. Although we focused only on the NF-κB- stemness gene pathway involving stemness genes ERBB2, SOX9, and MYC and WNT3 in our present study, we suspect other stemness genes or pathways may be involved as well in the process of IR-induced stemness [6, 11]. It will be well worth pursuing studies on this aspect of the BCSCs/iBCSCs challenge to RT.

RT plays a significant role in the management of early stage, locoregionally advanced, and metastatic breast cancer [38]. The combinatorial approach of RT with DSF/Cu, in which Cu^2+^ is endogenously present in tumors, we have described here provides a means to not only induce apoptosis of radioresistant pre-existing BCSCs, but also prevent the conversion of nonstem BC cells into radiation-induced BCSCs. It is worth noting that although addition of Cu^2+^ to DSF is definitely needed for *in vitro* depletion of BCSCs/iBCSCs (Fig. 1A, B, D) [27], exogenous copper should not be administered in conjunction with clinical use of DSF given the fact that many tumors including breast cancer tissues contain high levels of copper compared to normal tissues [39-44] *and* the beneficial effect of the copper depleting agent tetrathiomolybdate (TM) on reducing relapse in breast cancer patients[45]. Indeed, our *in vivo* data confirmed that exogenous copper gluconate administered orally (1mg/kg/day) with DSF(50 mg/kg/day) and IR (20Gy, once) was significantly less effective than DSF and IR without copper gluconate in inhibiting primary tumor growth and lung metastasis in the 4T1 mouse model system (data not shown). Therefore, one should be cautious on designing a clinical trial for cancer treatment using DSF and copper together.

In summary, our study offers a solid foundation for an immediate and practicable strategy of increasing the efficacy and curability of radiation therapy for breast cancer patients using a long established, safe and low cost drug DSF. Moreover, our study i) introduces the novel concept of blocking iBCSCs through targeting the NF-κB-stemness gene pathway, ii) reports the finding that IR, delivered locally to the primary mammary tumors, promotes distant lung metastasis in mice, and iii) contributes to a paradigm shift in understanding of the importance of targeting CSCs and blocking the formation of radiation induced CSCs in RT of cancers, and therefore leading to the next generation of radiotherapy.

## MATERIALS AND METHODS

### Cell culture

The human BC cell lines MDA-MB-231, MDA-MB-231-luc-D3H1, SUM149, UACC-812 and mouse BC cell line 4T1 were cultured in RPMI 1640 medium (Mediatech) supplemented with 2 mmol/L L-glutamine (Mediatech) and 10% fetal calf serum (FCS; Atlanta Biologicals). This cell culture medium is referred to as complete medium. Normal human mammary epithelial cells were purchased from Lifeline Cell Technology and cultured using MammaryLife Medium Complete Kit (Lifeline Cell Technology). All cells were cultured at 37°C in a 5% CO_2_ atmosphere. MDA-MB-231 and SUM149 cell lines were acquired from the Duke Comprehensive Cancer Center Cell Culture Facility. UACC-812 and 4T1 cell lines were purchased from the American Type Culture Collection (ATCC). The MDA-MB-231-luc-D3H1 cell line was obtained from Xenogen Corporation.

### Chemical reagents and antibodies

Tetraethylthiuram disulfide (disulfiram, DSF), Copper Chloride or Copper(II) D-gluconate (Cu), NF-κB inhibitor IMD-0354, and ROS inhibitor NAC were purchased from Sigma-Aldrich. DSF and IMD-0354 were reconstituted in DMSO for all *in vitro* experiments. DSF was reconstituted in olive oil for *in vivo* experiments. Cu was reconstituted in distilled water and NAC was reconstituted in RPMI 1640 medium for all experiments.

Antibodies for Western blotting: human and mouse HER2/ERBB2 (#2242) (1:1000 dilution)-, human and mouse c-myc (#9402) (1:1000 dilution)-, human cleaved PARP (#9541) (1:1000 dilution), human phosphorylated (p)-p38 MAPK (#9211) (1:1000 dilution)-, human p38 MAPK (#9212) (1:1000 dilution)-, human p-AKT (#9271) (1:1000 dilution), human AKT (#4685) (1:1000 dilution), and human and mouse β-actin (#4970) (1:2000 dilution)-specific rabbit monoclonal antibodies (mAbs) and goat anti-rabbit IgG, HRP-linked antibody (#7074) (1:2000) were purchased from Cell Signaling Technology. Human and mouse SOX9-specific rabbit antibody (Ab) (ab26414) (1μg/mL) was purchased from Abcam. All antibodies were diluted in Tris Buffered Saline with 0.1% Tween® 20 (TBST) containing 5% nonfat dry milk plus 2% bovine serum albumin (BSA). All dilutions were prepared immediately before use.

Antibodies for IHC staining: mouse Neu-specific rabbit Ab (#sc-284) (1:250 dilution) and mouse SOX9-specific rabbit Ab (#sc-20095) (1:200 dilution) were purchased from Santa Cruz Biotechnology. Mouse c-myc-specific rabbit mAb (#9402) (1:500 dilution) was purchased from Cell Signaling Technology. Mouse WNT3-specific rabbit Ab (#ab32249) (1μg/mL) was purchased from Abcam. All antibodies were diluted in TBST containing 5% normal horse serum and 1% BSA. All dilutions were prepared immediately before use. D-Luciferin Firefly, potassium salt was purchased from Xenogen. The Dual-Glo™ Luciferase Assay System, pGL4.32[luc^2^P/NF-κB-RE/Hygro]vector, and pRL-SV40 vector were purchased from Promega.

### Animals

Six week old NOD/SCID female mice were purchased from Taconic and 6 week old BALB/c female mice were purchased from the Massachusetts General Hospital COX7 animal facility. All the animal studies have been approved by the Institutional Animal Care and Use Committee.

### Flow cytometry and cell sorting

Cells were collected and were then incubated with ALDEFLUOR® reagent (Stem Cell Technologies), with or without the ALDH inhibitor DEAB according to the manufacturer's instructions. ALDH^neg^ cells were sorted using a BD Biosciences FACSAria II cell sorter.

### Irradiation (IR)

*In vitro* fractionated IR was performed on cells plated in 6-well plates at a density of 2x10^5^ cells/well in 2 mL of complete medium at 3.75Gy daily for 5 days unless otherwise specified. *In vitro* a single dose of IR was performed on cells plated in 6-well plates at a density of 2x10^5^ cells/well in 2 mL of complete medium at 10Gy. *In vivo,* a single dose of 20Gy was delivered locally to each mouse tumor. The Mark I Model 30 Cesium Irradiator (JL Shepherd and Associates) was used for all IR experiments in this study.

### Mammosphere formation

Cells were plated in 6-well plates at a density of 2x10^5^ cells/well in 2 mL of complete medium and received fractionated **irradiation**. Twenty-four hours after irradiation, cells were treated with DSF/Cu (2.5 μM/1 μM) or 1 μM NF-κB inhibitor IMD0354 for an additional 24 hours. Mammosphere formation was performed by seeding the cells (1000 cells/well) in a 24-well ultra-low adherent plate (Corning Incorporated) in 500 μL of mixed medium containing 32% MethoCult medium, 20% MammoCult basal human medium with a final concentration of 2% MammoCult proliferation supplements (STEMCELL Technologies), and 48% DMEM supplemented with final concentrations of 100 pg/mL EGF, 50 ng/mL bFGF, 5 ng/mL stem cell factor, 1×10^−6^ M hydrocortisone, and 5 μg/mL insulin. The cells were cultured at 37°C in a 5% O_2_ and 5% CO_2_ humidified atmosphere for 14 days. Mammosphere pictures were taken using a Zeiss Inverted Fluorescence Microscope on day 10 or 14. Numbers of mammosphere were count on day 14. Mammosphere formation was also performed by seeding (1000 cells/well) in a 24-well ultra-low adherent plate of the Fluorescence Activated Cell Sorting (FACS) sorted ALDH^neg^ cells or cells from a single cell suspension collected from a disaggregated tumor.

### Transfection with siRNA

MDA-MB-231 and SUM149 cells were plated in 12-well plates at a density of 4x10^4^ cells/well and 3.2x10^4^ cells/well in 0.5mL of complete medium, respectively. Cells were transfected with 100 nmol Signalsilence NF-κB p65 siRNA (#6261; Cell signaling Technology) using the X-tremeGENE siRNA Transfection Reagent (Roche) following the manufacturer's instructions. SignalSilence Control siRNA (#6201; Cell Signaling technology) was used as a control. Cells were then incubated for 48 hours before initiating specified treatments.

### Western blotting analysis

Cells were plated in 6-well plates at a density of 2x10^5^ cells/well in 2 mL of complete medium and were treated as indicated. Cells were collected and lysed in lysis buffer (10 mM Tris–HCl (pH 8.2), 1% NP40, 1 mM EDTA, 0.1% BSA, 150 mM NaCl) containing 1/50 (vol/vol) of a protease inhibitor cocktail (Calbiochem), or proteins were extracted from frozen mouse tumors by homogenization in the presence of 1 mL of lysis buffer. Western blotting for signaling-related proteins and proteins of stemness genes was carried out as described [46]. The investigators who analyzed the samples from the tumors were blinded to the type of treatment received by the mice.

### Cell proliferation and MTT assay

Cells were plated in triplicate in 96-well plates at a density of 2.5×10^3^ cells/well in 100 uL of complete medium and were treated as indicated. Cell proliferation was evaluated by an MTT assay at the indicated time point. MTT assays were carried out as described [46].

### Real-Time qRT-PCR

Cells were plated in 6-well plates at a density of 2×10^5^ cells/well in 2 mL of complete medium and were treated as indicated. Total RNA from all *in vitro* experimental samples or frozen mouse tumors was isolated using TRIzol (Life Technologies), and was reverse transcribed using M-MLV reverse transcriptase (Life Technologies) according to the manufacturer's instructions. All samples within each experiment were reverse transcribed at the same time. The resulting cDNA was diluted to a concentration of 100 ng/mL in nuclease-free water and was stored in aliquots at –20°C until use. Real-time qPCR using cDNA as a template with SYBR green detection was performed using a LightCycler 96 system (Roche) with FastStart SYBR Green Master Mix (Roche) and primers for each gene as shown in Supplementary Table S4. Appropriate no-RT and non-template controls were included in each 96-well PCR reaction, and dissociation analysis was performed at the end of each run to confirm the specificity of the reaction. Each gene expression level was normalized to mRNA levels of the housekeeping gene r18S. Fold changes of each gene's expression was compared to that of the untreated sample using the 2(-Delta Delta C(T)) method [47].

### Dual-Glo luciferase assay

MDA-MB-231 cells were plated in 12-well plates at a density of 4×10^4^ cells/well in 0.5 mL of complete medium and cultured for 24 hours, the pGL4.32[luc2P/NF-κ^3^ba;B-RE/Hygro] and pRL-SV40 vectors were co-transfected into adherent cells. After 24 hours, the transfected cells were irradiated with a single dose of 10 Gy and were then treated with DSF/Cu (2.5 μM/1 μM) or 1 μM IMD-0354 for 24 hours. A dual luciferase assay was then performed with these cells using the Dual-Glo luciferase assay kit according to the manufacturer's instructions. NF-κB luciferase activity, normalized to Renilla luciferase activity, was expressed relative to that of the untreated cells, which was set at 1.0.

### *In vivo* tumorigenicity assay

*In vitro,* MDA-MB-231-luc-D3H1 cells were fraction-irradiated at 3.75 Gy/day for 5 days and were cultured for 24 hours followed by an additional 24 hours with or without the treatment of DSF/Cu (2.5 μM/1 μM). Three groups of NOD/SCID mice (n=4) were injected i.p. with either untreated MDA-MB-231-luc-D3H1 cells, irradiated MDA-MB-231-luc-D3H1 cells treated with DSF/Cu, or only irradiated MDA-MB-231-luc-D3H1 cells. The same number of cells was used for each group, with 2x10^6^ cells in 25 μL RPMI 1640 and 25 μL Matrigel injected i.p. in each mouse. Tumorigenicity measured as tumor growth was monitored in each mouse every 3-4 days by bioluminescence image (BLI) and tumor burden quantitated as photons/second.

### *In vivo* syngeneic tumor model

Mouse BC 4T1 cells (5x10^5^ cells/mouse) were implanted subcutaneously in the thigh of right hind leg of BLAB/c mice. Body weight and tumor volume were measured twice per week. Tumor volume was measured by digital calipers. Treatments were initiated on day 7 when the tumor developed and had an approximate diameter of 5 mm. Mice were divided into 4 groups (n=7) using a stratified randomization strategy, such that the difference of mean tumor volumes was not statistically significant between each group (P>0.5). Mice in group 1 were treated with olive oil (vehicle control, 200 μL) orally from day 7 to day 14. Mice in group 2 were treated with a 20Gy irradiation on day 10. Mice in group 3 were treated with oral administration of DSF (50 mg/kg/day) from day 7 to day 14. Mice in group 4 were treated with a 20Gy irradiation on day 10 and oral administration of DSF (50 mg/kg/day) from day 7 to day 14. All oral administration was given by an oral gavage using an 18 gauge plastic feeding tube (Solomon Scientific). When tumor diameters from untreated mice reached 1.5 cm, all mice were sacrificed. Primary tumors and lungs were collected for further analysis.

### Samples preparations from primary mouse tumors

For mammosphere formation assay: a non-necrotic primary tumor tissue specimen from each mouse was collected at the time of sacrifice, minced into 3x3 mm pieces and digested with Collagenase IV (1mg/mL PBS)(Worthington Biochemical Corp.) at 37°C for 1 hour. A single cell suspension was obtained by filtering the digested tumor through a 40μM cell strainer.

For total RNA and protein extractions: a non-necrotic primary tumor tissue specimen from each mouse was collected at the time of sacrifice, minced (3x3 mm) and stored at -80°C for total RNA or protein extraction.

For IHC staining: the remaining non-necrotic tumor tissue specimen from each mouse was collected at the time of sacrifice and formalin-fixed and paraffin-embedded (FFPE). FFPE blocks were sectioned using MICROM HM325 Rotary Microtome (Thermo Fisher Scientific Inc).

### Analysis of areas of lung metastasis

At the time of sacrifice, mouse lungs were collected as described above for histological analysis of lung metastasis. Four micron-thick formalin-fixed paraffin-embedded (FFPE) sections of mouse lung tissue from treated BALB/c mice bearing 4T1 cell tumors were stained with Mayer's Hematoxylin (Sigma-Aldrich) and Eosin Y solution (Sigma-Aldrich) according to the manufacturer's instructions. The areas of the metastatic nodules in 10 randomly selected high-power fields (200x magnification) per section were measured and calculated by the SPOT Advanced Imaging software (Diagnostic Instruments). The research fellow who analyzed the lungs was blinded to the type of treatment received by the mice.

### IHC staining

Four micron-thick tissue sections were deparaffinized and subjected to antigen retrieval. Following incubation with blocking buffer, slides were incubated with anti-Neu, -SOX9, -c-myc, and -WNT3 Abs overnight. Sections were then rinsed with TBST, followed by 45-minute incubation with the EnVision™+ Dual Link System-HRP goat anti-rabbit Ig (Dako). The detection of staining signals was performed with the DAB Peroxidase Substrate Kit from Dako, and the sections were counterstained with hematoxylin. Staining results of stemness gene protein were analyzed by two investigators who were blinded to the type of treatment received by the mice, by counting the positive cells in 10 randomly selected high-power (200x) fields. Complete membrane staining was considered as positive for Neu expression; nuclear staining was considered as positive for SOX9 and c-myc expression, and cell membrane and cytoplasm staining were considered as positive for WNT3 expression.

### Statistical analysis

For *in vivo* tumor volumes, estimation of the treatment group specific mean responses over time and their comparisons were performed by using longitudinal general linear mixed effects model with compound symmetry error covariance structure. The subject level intercepts were random, and both time effect and time x treatments interactions were considered fixed effects. The longitudinal treatment effects were compared by comparing the treatment specific slopes by linear contrast. For all other data analysis, averages, standard deviations, and unpaired *t*-tests were calculated using Microsoft Excel. *In vitro* data shown represent the mean ± SD of the results obtained in at least three independent experiments. *In vivo* data shown represent the mean ± SD of the results obtained in each group. Differences between groups were considered significant when the *P* value was *<* 0.05.

## SUPPLEMENTARY FIGURES AND TABLES




