# Supplementary material for: Blocking the formation of radiation–induced breast cancer stem cells
Source: Oncotarget. 2014 May 20;5(11):3743–55. doi: 10.18632/oncotarget.1992 (PMC4116517; doi:10.18632/oncotarget.1992)
Supplement: Supplementary file 1 [file oncotarget-05-3743-s001.pdf]

Blocking the formation of radiation–induced breast cancer stem cells

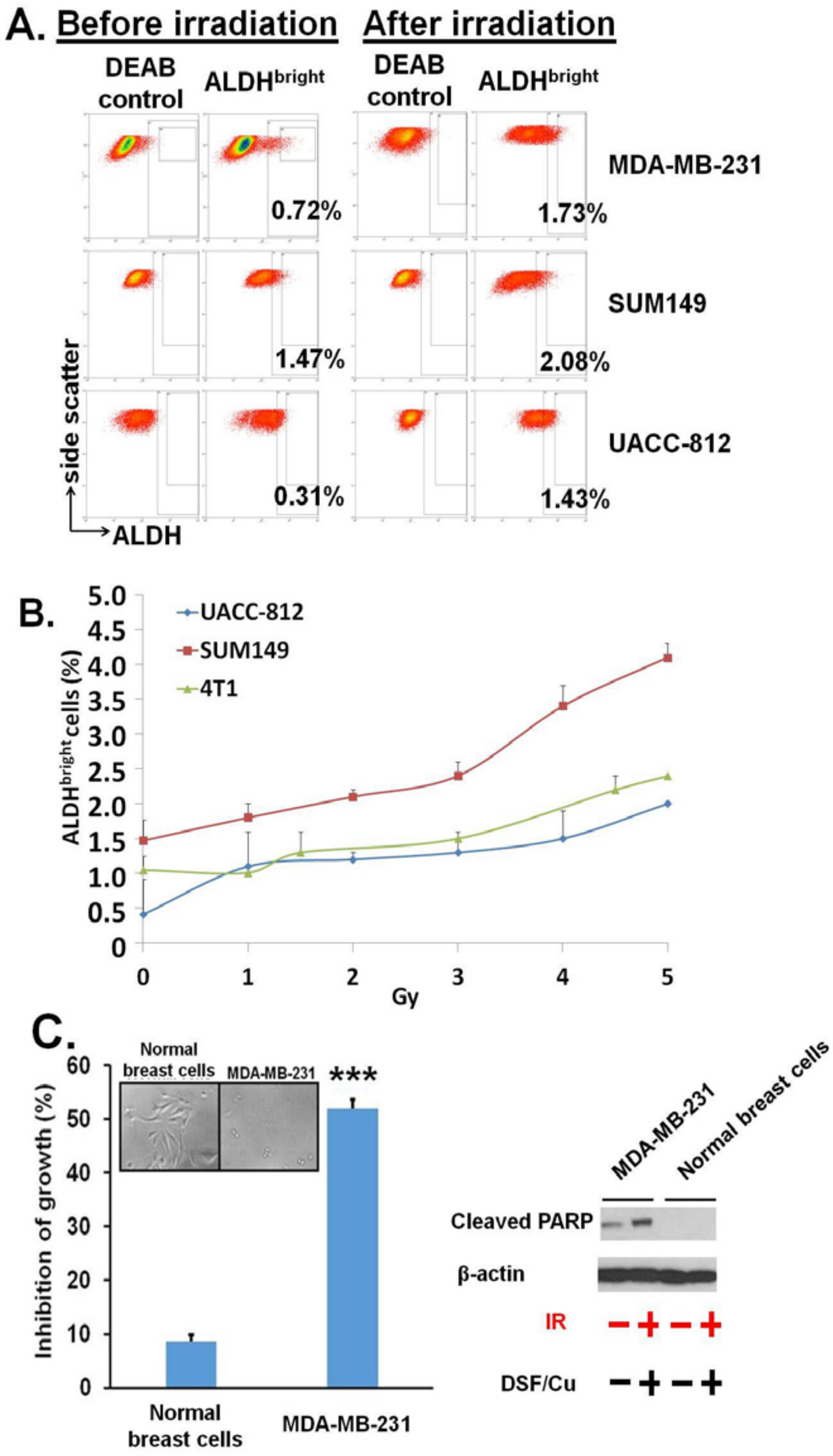

Supplementary Figure S1: IR-induced iBCSCs *in vitro* measured by an increased percentage of ALDH^{bright} cells and mammosphere formation . Human BC cell lines MDA-MB-231, SUM149, and UACC-812 were fraction-irradiated at a fixed dosage (3.75 Gy/day x 5 days). The cells were then cultured for an additional 48 hours and analyzed by flow cytometry using ALDEFLUOR to detect ALDH^{bright} cells as indicated**.** DEAB, the specific inhibitor of ALDH1/3 isoforms, was used to establish the baseline fluorescence of each cell line as a gating reference standard of the ALDH^{neg} population in each sample (**A**). Human BC cell lines were fraction-irradiated at indicated doses daily for 5 days and followed by an additional 48 hours culture. The percent of ALDH^{bright} cells was then analyzed using ALDEFLUOR assay. All of the experiments were performed in duplicate and repeated twice and the means ± SD are shown (**B**). Normal human mammary epithelial cells (referred to as normal breast cells) and MDA-MB-231 cells were seeded in a 96-well plate and treated with DSF/Cu (0.03µM/1µM) for 48 hours. Pictures of the cells were taken using a Zeiss Inverted Fluorescence Microscope and cell viability was analyzed by MTT assays. Inhibition of cell growth was calculated using untreated cells as a 100% cell growth reference. The experiments were performed three independent times in triplicate and the means ± SD are shown. *** indicates $p<0.001$. Normal human mammary epithelial cells and MDA-MB-231 cells were treated with DSF/Cu (10µM /1µM) for 3 hours and then a single dose of 10 Gy IR 10 minutes before lysing cells. The cell lysates were analyzed by Western blotting for detection of alterations in cleaved PARP, a marker of apoptosis. β-actin was utilized as a loading control (**C**).

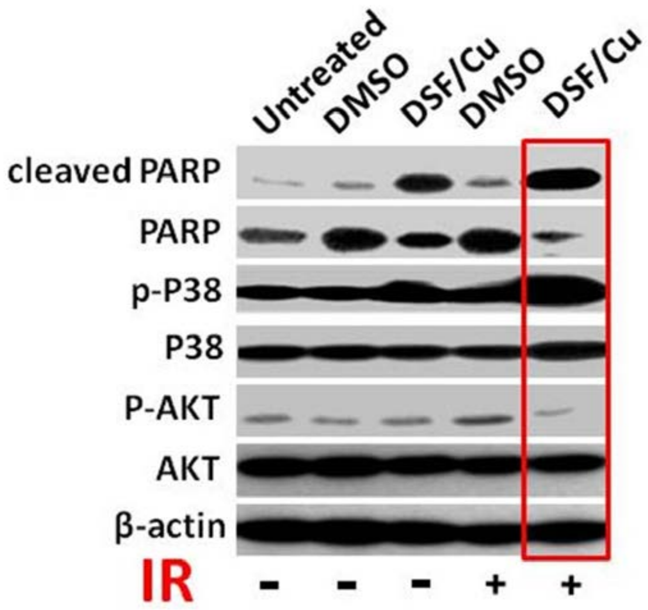

Supplementary Figure S2: DSF/Cu and IR enhanced the upregulation of p38 MAPK and downregulated AKT signaling resulting in increased apoptosis in breast

cancer cells. UACC-812 cells were treated with DSF/Cu (10µM /1µM) for 3 hours and then a single dose of 10 Gy IR 10 minutes before lysing cells. The cell lysates were analyzed by Western blotting for detection of alterations in cleaved PARP, p38 MAPK, phosphorylated (p)-p38, AKT, and p-AKT. As indicated, increased apoptosis (cleaved PARP), upregulated p-p38, and downregulated p-AKT levels were found in cells treated with IR+DSF/Cu, in comparison to cells treated only with DSF/Cu or IR+DMSO.

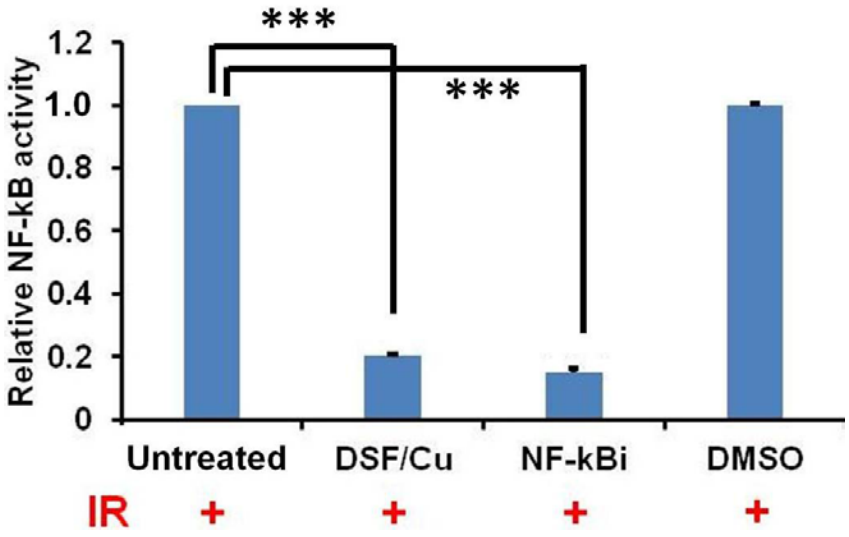

Supplementary Figure S3: Inhibition of NF-κB-driven luciferase activity by DSF/Cu. MDA-MB-231 cells were transfected with NF-κB dual reporter system vectors. After 24 hours, the transfected cells were irradiated with 10 Gy of a single dose and then immediately treated with DSF/Cu (2.5µM/1µM) or the NF-κB inhibitor IMD-0354 (1µM) for 24 hours. A dual luciferase assay was then performed. NF-κB-driven luciferase activity, normalized to Renilla luciferase activity, was expressed relative to that of the untreated cells, which was set at 1.0. The experiments were performed in duplicate and repeated twice and the means ± SD are shown. *** indicates $p<0.001$.
