# Supplementary material for: Blocking the formation of radiation–induced breast cancer stem cells
Source: Oncotarget. 2014 May 20;5(11):3743–55. doi: 10.18632/oncotarget.1992 (PMC4116517; doi:10.18632/oncotarget.1992)
Supplement: Supplementary file 2 [file oncotarget-05-3743-s002.pdf]

Supplementary Table S1. Potential NF-kB binding sites in the promoter regions of WNT3. A.

Potential NF-kB binding sites in the promoter regions of mouse WNT3.

Gene	TF	TF-BS Position (NC_)
WNT3	NF-κB	103620251
WNT3	NF-κB	103632344
WNT3	NF-κB	103632963
WNT3	NF-κB	103632965
WNT3	NF-κB	103639373
WNT3	NF-κB	103641492
**Binding Position**	**Strand**	**Binding Sequence**
chr11: 103620244-103620257	-	AGGGGGCGCTCCTG
chr11: 103632337-103632350	-	GGGGAAATCTCCCA
chr11: 103632956-103632969	-	GGGGATTTCCCCCC
chr11: 103632960-103632969	-	GGGGATTTCC
chr11: 103639368-103639377	-	GGGGCCTTCC
chr11: 103641486-103641497	-	GGGGAGACCCCA

B. Potential NF-kB binding sites in the promoter regions of human WNT3.

Gene	TF	TF-BS Position (NC_)
Wnt3	NF-κB	44889955
Wnt3	NF-κB	44892076
Wnt3	NF-κB	44898658
Wnt3	NF-κB	44898659
Wnt3	NF-κB	44899319
**Binding Position**	**Strand**	**Binding Sequence**
chr17: 44889949-44889961	+	AGGGGACACTCCA
chr17: 44892071-44892081	+	TGGGGCCTTCC
chr17: 44898653-44898663	+	TGGGGATTTCC
chr17: 44898653-44898665	+	TGGGGATTTCCGC
chr17: 44899312-44899326	+	GGAGGAAATCTCCGA

All potential binding sites were found on:
http://www.sabiosciences.com/chipqpcrsearch.php?app=TFBS

Supplementary Table S2. Decreasing *in vitro* stemness genes of mRNA expression after irradiation and DSF/Cu treatment.

Cell lines	MDA-MB-231			SUM149		
gene / Treatment	ERBB2	SOX9	MYC	ERBB2	SOX9	MYC
IR	4.45	2.42	1.38	8.25	5.17	3.77
IR + DSF/Cu	2.58	-1.22	-2.75	5.88	3.92	2.46
IR + NF-kBi	-1.11	-1.28	-5.26	4.89	2.54	2.03
IR + ROSi	4.92	2.39	1.32	9.78	4.84	3.05
IR + DSF/Cu + ROSi	2.89	-1.19	-2.96	5.43	3.96	2.87
IR + NF-kBi + ROSi	-1.07	-1.24	-5.31	4.75	2.63	2.12
IR + DMSO	4.63	2.41	1.36	9.51	5.80	3.76
IR + control siRNA	4.52	2.57	1.52	8.31	5.04	3.54
IR + p65 siRNA	2.31	-1.12	-2.83	5.42	3.87	2.51
IR + p65 siRNA + ROSi	2.42	-1.14	-2.71	5.37	3.94	2.61
IR + DSF/Cu vs. IR	-1.72	-2.95	-3.83	-1.40	-1.32	-1.53

Cell lines	4T1		
gene / Treatment	ERBB2	SOX9	MYC
IR	9.51	3.99	2.83
IR + DSF/Cu	6.13	1.44	1.44
IR + NF-kBi	4.84	1.43	1.85
IR + ROSi	8.00	4.25	2.58
IR + DSF/Cu + ROSi	6.61	1.85	1.61
IR + NF-kBi + ROSi	4.92	1.52	1.88
IR + DMSO	8.00	4.92	2.87
IR + DSF/Cu vs. IR	-1.55	-2.77	-1.97

Fold changes of stemness genes of treated cells were compared with that of untreated cells except the last row showed fold changes between IR + DSF/Cu and IR only.
Positive number indicates fold increase and negative number indicates fold decrease. Each gene expression level was normalized to mRNA levels of the housekeeping gene r18S. Fold changes of each gene's expression are compared to that of the untreated sample using a 2(-Delta Delta C(T)) method: $\Delta\Delta CT=(CT_{Target}-CT_{r18S})_{Time\ x} - (CT_{Target} - CT_{r18S})_{Time\ 0}$ [47].

Supplementary Table S3. Decreasing *in vivo* stemness genes of mRNA expression after irradiation and DSF treatment.

Detection	Early (Day 15)				Late (Day 29)			
gene / Treatment	ERBB2	SOX9	MYC	WNT3	ERBB2	SOX9	MYC	WNT3
DSF	-6.24	-1.88	-3.21	-4.42	1.51	1.22	-2.73	-2.28
IR	1.41	4.52	1.75	1.59	2.00	2.31	1.21	1.33
IR + DSF	-2.94	-1.15	-2.86	-3.93	1.22	1.20	-3.41	-3.95
IR + DSF vs. IR	-4.13	-5.18	-5.01	-6.25	-2.43	-1.92	-4.13	-5.25

Fold changes of stemness genes of tumors from treated groups were compared with that from vehicle control group, except the last row showed fold changes between IR + DSF and IR only. Positive number indicates fold increase and negative number indicates fold decrease.

Supplementary Table S4. Primers for stemness genes for Real-Time qRT-PCR assays.

		5' -3' sequence
human	ERBB2 Forward	AGCCGCGAGCACCCAAGT
	ERBB2 Reverse	TTGGTGGGCAGGTAGGTGAGTT
	SOX9 Forward	GGGAAGGCCGCCCAGGGCGA
	SOX9 Reverse	TGCCTTGCCCGACTGCAGTTCT
	MYC Forward	CGGAACTCTTGTGCGTAAGG
	MYC Reverse	CTCAGCCAAGGTTGTGAGGT
mouse	ERBB2 Forward	CCCAGATCTCCACTGGCTCC
	ERBB2 Reverse	TTCAGGGTTCTCCACAGCACC
	SOX9 Forward	GAGCCGGATCTGAAGAGGGA
	SOX9 Reverse	GCTTGACGTGTGGCTTGTTC
	MYC Forward	TAACTCGAGGAGGAGCTGGA
	MYC Reverse	GCCAAGGTTGTGAGGTTAGG
	WNT3 Forward	GGAGAAACGGAAGGAGAAATG
	WNT3 Reverse	GAGAGACGTTAGTTGAGAAAGAAGC
Housekeeping gene	r18S Forward	TCAAGAACGAAAGTCGGAGG
	r18S Reverse	GGACATCTAAGGGCATCACA
